# Impact of dapagliflozin on metabolic phenotype, hormone levels, and fertility in female mice after prolonged high-fat diet

**DOI:** 10.3389/fendo.2024.1457268

**Published:** 2025-01-21

**Authors:** Xiaolin Chen, Zhuoni Xiao, Na Dai, Mingxia Fan

**Affiliations:** ^1^ Department of Endocrinology, Renmin Hospital of Wuhan University, Wuhan, China; ^2^ Reproductive Medical Center, Renmin Hospital of Wuhan University, Wuhan, China; ^3^ Center for Animal Experiment, Renmin Hospital of Wuhan University, Wuhan, China

**Keywords:** sodium-glucose cotransporter 2, metabolism, insulin resistance, reproductive function, high-fat diet

## Abstract

**Introduction:**

A long-term high-fat diet (HFD) cause obesity and infertility through hypothalamic inflammation and insulin resistance, leading to metabolic abnormalities and ovulation dysfunction. The sodium-glucose cotransporter 2 inhibitors (SGLT2i) have emerged as a treatment for type 2 diabetic patients, regulating adipose tissue metabolism, hypothalamic inflammation, and ovulation in women with polycystic ovary syndrome (PCOS). The study aimed to investigate the pharmacological effects of dapagliflozin on improving insulin resistance, energy metabolism, sex hormones, and fertility in female mice following prolonged consumption of HFD.

**Methods:**

At 6 weeks of age, female mice were fed a HFD and treated with dapagliflozin. Serum hormone concentrations and inflammatory factors in mice aged 28 weeks or 38 weeks were quantified using ultrasensitive enzyme-linked immunosorbent assays (ELISAs). Metabolic parameters were also assessed and documented at different stages of the experiment. At 34 weeks of age, half of the experimental mice in each of the four groups fed with standard chow were mated with male mice. Pregnancy rate, abortion rate, pregnancy-related deaths, and perinatal outcomes were systematically recorded.

**Results:**

After 16 weeks of HFD feeding, dapagliflozin significantly attenuated visceral fat deposition, weight gain, glucose intolerance, and insulin resistance induced by the diet. However, these effects diminished after 32 weeks. Unexpectedly, neither HFD nor dapagliflozin treatment elicited any significant changes in serum IL-6 and TNFα levels. Throughout the experiment period, dapagliflozin exhibited favorable effects on reproductive function along with insulin sensitivity and luteinizing hormone (LH) release from the pituitary gland.

**Discussion:**

In conclusion, this study demonstrates that dapagliflozin alleviated HFD-induced reproductive dysfunction independently of obesity, peripheral tissue insulin resistance, and systemic inflammation, suggesting its potential as a promising treatment for diet-related ovulation disorders.

## Introduction

High-fat diet (HFD) intake is a major risk factor for obesity, which in turn leads to an increased incidence of infertility, maternal mortality, pregnancy complications, and adverse birth outcomes, as well as metabolic alterations in offspring (1–4). In our previous study, we found that 22 weeks of HFD intake resulted in microglia activation, hypothalamic inflammation, obesity, and ovulatory dysfunction in mice. However, treatment with dapagliflozin was effective in alleviating these HFD-related impairments (5). Our research team also demonstrated that dapagliflozin improved metabolic disorders, normalized the estrous cycle, and restored pulsatile luteinizing hormone (LH) secretion and ovulation in melanocortin-4 receptor knockout (MC4R KO) obese mice (6). While our previous studies have primarily focused on the metabolic and ovulatory disorders caused by HFD in mice and the beneficial effects of sodium–glucose cotransporter type 2 inhibitors (SGLT-2i), there has been comparatively less attention on pregnancy outcomes and perinatal consequences as well as their impact on offspring.

According to the latest statistics, the infertility rate among Chinese couples of reproductive age has risen to 18% (7). Among infertile couples, solely female factors account for more than 40% of cases, with approximately 40% of these being attributed to ovulation disorders (8, 9). Polycystic ovary syndrome (PCOS) is the most prevalent neuroendocrine metabolic disease, characterized by hyperandrogenism, anovulation, polycystic ovarian morphology, obesity, insulin resistance, and chronic systemic inflammation (10). In China, it affects approximately 7.8% of women of reproductive age (11). In addition, there has been a significant increase in the incidence of PCOS associated with HFD consumption (12). Current clinical trials and basic research indicate a potential role for SGLT2i in treating obesity-related PCOS (13–17). Thus, it is crucial to initiate further studies to investigate the long-term effects of HFD consumption on the metabolic status, estrous cycles, and sex hormone levels in female mice. Additionally, it is important to determine the impact of SGLT2i on pregnancy outcomes as well as perinatal health and offspring development.

In the current study, our objective is to investigate the impacts of prolonged consumption of HFD and treatment with SGLT2i on metabolic and reproductive phenotype in female mice. Additionally, we aim to evaluate the potential role of SGLT2i pretreatment in the pregnancy outcomes, perinatal health, and offspring development.

## Material and methods

### Animals

Five-week-old female C57BL/6J mice were purchased from the Experimental Animal Center and housed at the Animal Center of the Renmin Hospital of Wuhan University. Four-six mice were housed per cage with free access to food and water. The study was approved by the Ethics Committee of Wuhan University, China (IRB approval number: WDRM 20210612) and followed ARRIVE guidelines 2.0. Mice were acclimated for 1 week and then randomized into four groups: chow (n = 8), chow +dapagliflozin (1mg/kg) (n = 8), high-fat diet (HFD, n = 12), and HFD + dapagliflozin (1mg/kg) (n = 12). The standard diet was purchased from Beijing Keao Xieli Feed Co., Ltd (340 kcal/100g, 11.85% from fat, 65.08% kcal from carbohydrate, and 23.07% kcal from protein). The HFD was purchased from Jiangsu Synergy Pharmaceutical Bioengineering Co., Ltd (D12492, 524 kcal/100 g, 60% kcal from fat, 20% kcal from carbohydrate, and 20% kcal from protein). Dapagliflozin was administered in the diet based on daily food intake (3~ 5g for mice). Vaginal cytology using vaginal smears taken between 14:00 and 16:00 p.m. was used to determine the stage of the estrous cycle, including proestrus, estrous, and diestrus stages.

### Experimental design

**Figure 1:**
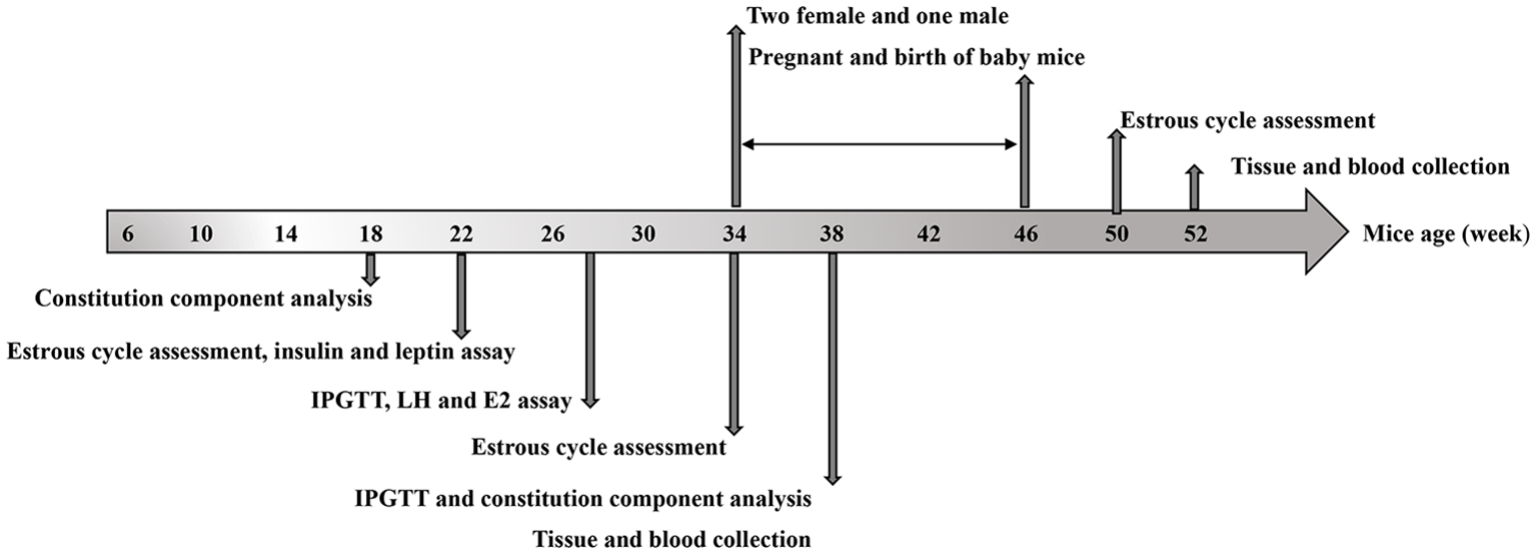
Experimental design and timeline: The timeline of HFD exposure, dapagliflozin treatment, biochemical tests, and tissue collection.

**Figure 1** shows the experimental timeline, including biweekly recording of mouse body weight, constitution component analysis at 18 or 38 weeks, assessment of estrous cycle at specific intervals, measurement of serum insulin and leptin levels at 22, 38, and 52 weeks, measurement of serum IL-6 and TNFα levels at 38 and 52 weeks, glucose tolerance test was tested at 28 and 37 weeks. At the age of 34 weeks old, half of the experimental mice in each of the four groups were mated with male mice (female: male = 2:1). Additionally, two of the groups fed with a HFD switched to standard chow. Vaginal plugs were checked daily, with an experimental endpoint established at eight weeks (ranging from 34 to 42 weeks of age), taking into account that the gestation period is approximately twenty-one days. Pregnancy outcomes including pregnancy rate, abortion rate, pregnancy-related deaths as well as perinatal outcomes such as number and weight of offspring and stillbirth were recorded. Mice were sacrificed after fasting for 8 hours at the age of 52 weeks during diestrus stage. The remaining half of mice were sacrificed at the age of 38 weeks.

### Measures of metabolic phenotype

Intraperitoneal glucose tolerance tests (IPGTT) were performed at 26-week and 38-week ages, while insulin tolerance tests (IPITT) were performed at 38 weeks of age. For IPGTT, mice fasted 10 hours before receiving a single dose of glucose (2g/kg). IPITT involved a single dose of insulin (0.6U/kg; Novo Nordisk) after a 6-hour fast starting at 08:00 h. Blood sugar levels were tested using the FR201BC glucometer (glucose oxidase method, MEDISAFE FIT, TERUMO, Japan). The homeostasis model assessment of the insulin resistance index (HOMA-IR) was calculated using the equation [fasting insulin concentration (mU/L) x fasting glucose concentration (mmol/L)]/22.5 for all mice. Body fat percentage and bone mineral density (BMD) were assessed using dual energy X-ray absorptiometry (Hologic, Discovery Wi, USA). At the end of the experiment, adipose tissues and various organs including liver, kidney, heart, ovarian, uterine and periovarian adipose tissue (OUA) were isolated and weighted individually. Ovarian, uterine, and periovarian adipose tissue were weighed together.

### ELISA for insulin, leptin, IL-1β, IL-6, TNFα and sex hormones

All blood samples were collected following an 8-hour fasting period. Insulin, leptin, IL-1β;, IL-6, TNFα, testosterone (T), and progesterone (P) levels were measured during diestrus stage. LH, estradiol 2 (E2), and P levels were measured during the proestrus, estrus, and diestrus stages, respectively. Ultrasensitive mouse enzyme-linked immunosorbent assays (ELISAs) kits from Fine Test in China were used for all measurement. The intra‐ and inter-assay precisions were both ≤ 10% CV.

### Histopathological analysis

The tissues were fixed in 10% v/v formalin for 24 h and embedded in paraffin. Sections (12 μm) were obtained using a cryostat (LEICA Microsystems, CM1860) and digitally scanned with a Pannoramic 250 FLASH scanner (3DHISTECH, Hungary).

For immunohistochemical staining, the paraffin sections were washed with PBS, incubated in blocking buffer, then treated with anti-SGLT2 (24654-1-AP, PTGLAB, 1:50) and anti-insulin receptor (GB113494, Servicebio, 1:1000) antibodies followed by a secondary antibody (HRP-goat antirabbit, 1:200) at 37°C. Slides were counterstained with 4’,6-diamidino-2-phenylindole dihydrochloride (DAPI, 1:200).

For immunofluorescence staining, paraffin sections were washed with PBS, blocked with bovine serum albumin, incubated overnight with primary antibodies (anti-insulin receptor, and anti-SGLT2), then incubated with a conjugated antirabbit secondary antibody and stained with DAPI dye.

The paraffin sections of ovary and adipose tissues were dewaxed with xylene, rehydrated with alcohol, dyed with hematoxylin and eosin, washed, sealed, and placed in a fume cabinet overnight.

### Statistical analysis

The data were shown as the means ± SD and analyzed using GraphPad Prism 9.0 software (GraphPad, CA, USA). Two-way and one-way ANOVA tests with Tukey *post hoc* analysis were used for parametric data, while a Chi-square test was used for non-parametric data. Statistical significance was set at *P* < 0.05.

## Results

### Changes in weight and body composition

**Figure 2:**
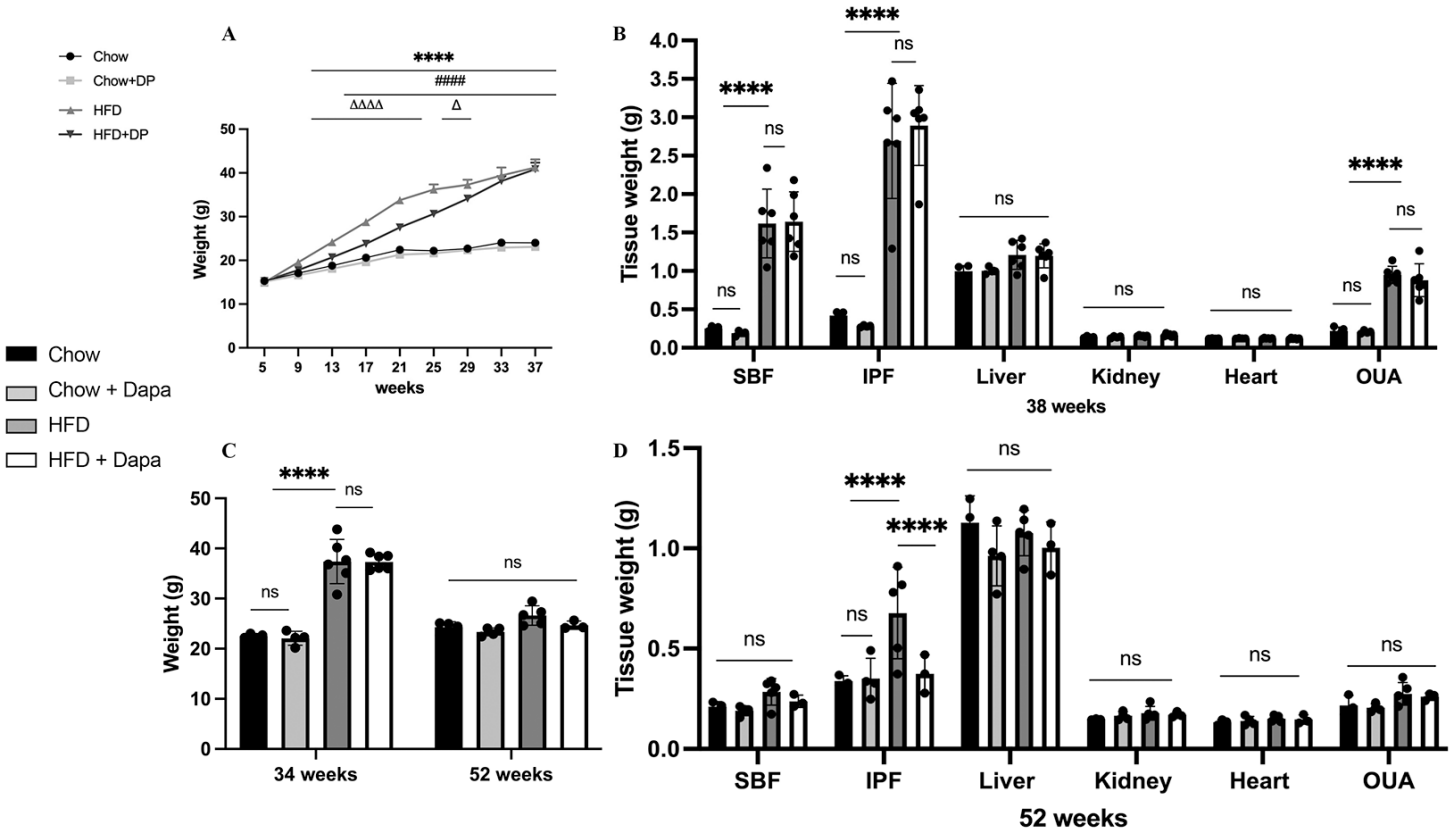
Body weight and tissue weight of mice. **(A)** Body weight; **(B)** Tissues weight of mice at 38 weeks of age. **(C)** Body weight at 34 weeks and 52 weeks of age, respectively; **(D)** Tissues weight of mice at 52 weeks of age. 34 weeks, n = 4-6/group, 38 weeks, n = 3-5/group. The data was analyzed using ANOVA with Tukey’s test. Shown are the mean SD. ^****^ *P* < 0.0001, HFD vs Chow or Chow + Dapa; ^####^ *P* < 0.0001, HFD vs HFD + Dapa; ^△^ *P* < 0.05, ^△△△△^ *P* < 0.0001, HFD + Dapa vs Chow or Chow + Dapa. ns, no significance. SBF, subcutaneous fat; IPF, retroperitoneal and mesenteric fat; OUA, ovarian, uterine and peri-ovarian adipose tissue.

After weaning and adaptive breeding for one week, C57BL/6J female mice were divided into groups fed with either a HFD or chow diet, with or without dapagliflozin. Body weights were measured every four weeks. At 38 weeks old, mice on the HFD showed weight gain, but dapagliflozin treatment partially attenuated this effect before 29 weeks (*P* < 0.0001). However, following 27 weeks of exposure to HFD, the mice exhibited resistance to dapagliflozin treatment, leading to no statistically significant difference in weight between the two groups subjected to HFD (**Figure 2A**). The weights of subcutaneous fat (SBF), retroperitoneal and mesenteric fat (IPF), and OUA in HFD-fed mice were heavier than those in chow-fed mice, with no difference between HFD-fed mice with or without dapagliflozin treatment (*P >*0.05). The weights of the liver, kidney, and heart did not differ among the four groups (**Figure 2B**). **Figure 2C** demonstrated weight of mice at 34 weeks and 52 weeks of age. The results showed no significant difference in body weight among the four groups at 52 weeks of age. The weights of the liver, kidney, heart, SBF, and OUA showed no difference among the groups, but HFD-fed mice had significantly heavier IPF compared to other groups (**Figure 2D**).

**Figure 3:**
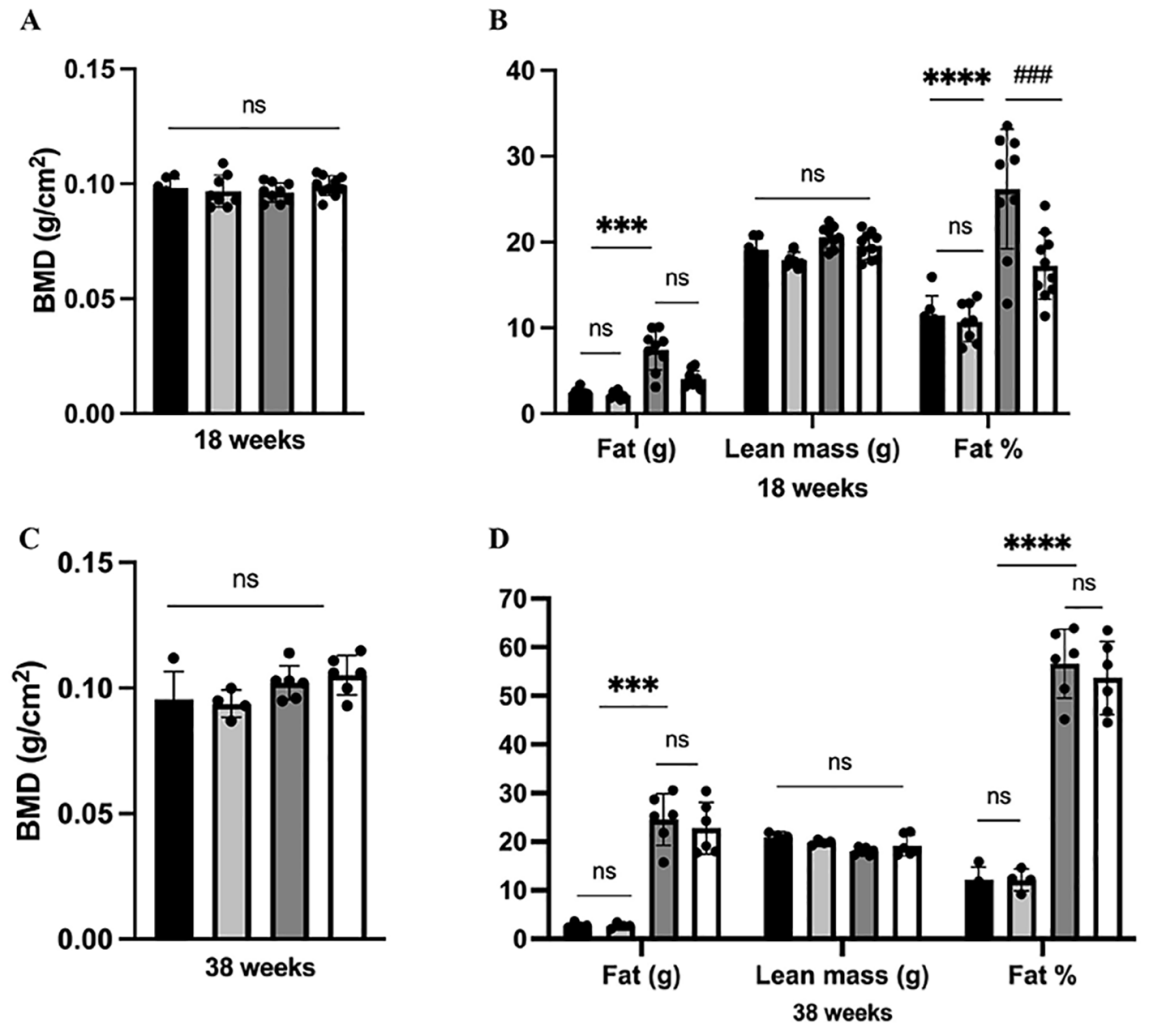
Analysis of body composition in mice. **(A)** BMD at 38 weeks of age; **(B)** Body composition at 18 weeks of age; **(C)** BMD at 38 weeks of age; **(D)** Body composition at 38 weeks of age. 18 weeks, n = 8-12/group, 38 weeks, n = 4-6/group. The data was analyzed using ANOVA with Tukey’s test. Shown are the mean SD. ^***^ *P* < 0.001, ^****^ *P* < 0.0001, HFD vs Chow or Chow + Dapa; ^###^ *P* < 0.001, HFD vs HFD + Dapa; ns, no significance, BMD, bone mineral density.

Moreover, our finding demonstrated that neither HFD nor dapagliflozin had any impact on BMD or lean mass after 13 weeks or 33 weeks of exposure (*P >*0.05) (**Figures 3A, C**). Increased body fat is a characteristic of obesity. In line with the analysis of body weight, a significant rise in fat weight and percentage of fat was observed after 12 weeks of HFD exposure; Dapagliflozin treatment also significantly reduced the HFD-induced increase in body fat (**Figure 3B**). However, these beneficial effects of dapagliflozin were not observed following 32 weeks of HFD exposure (**Figure 3D**).

**Figure 4:**
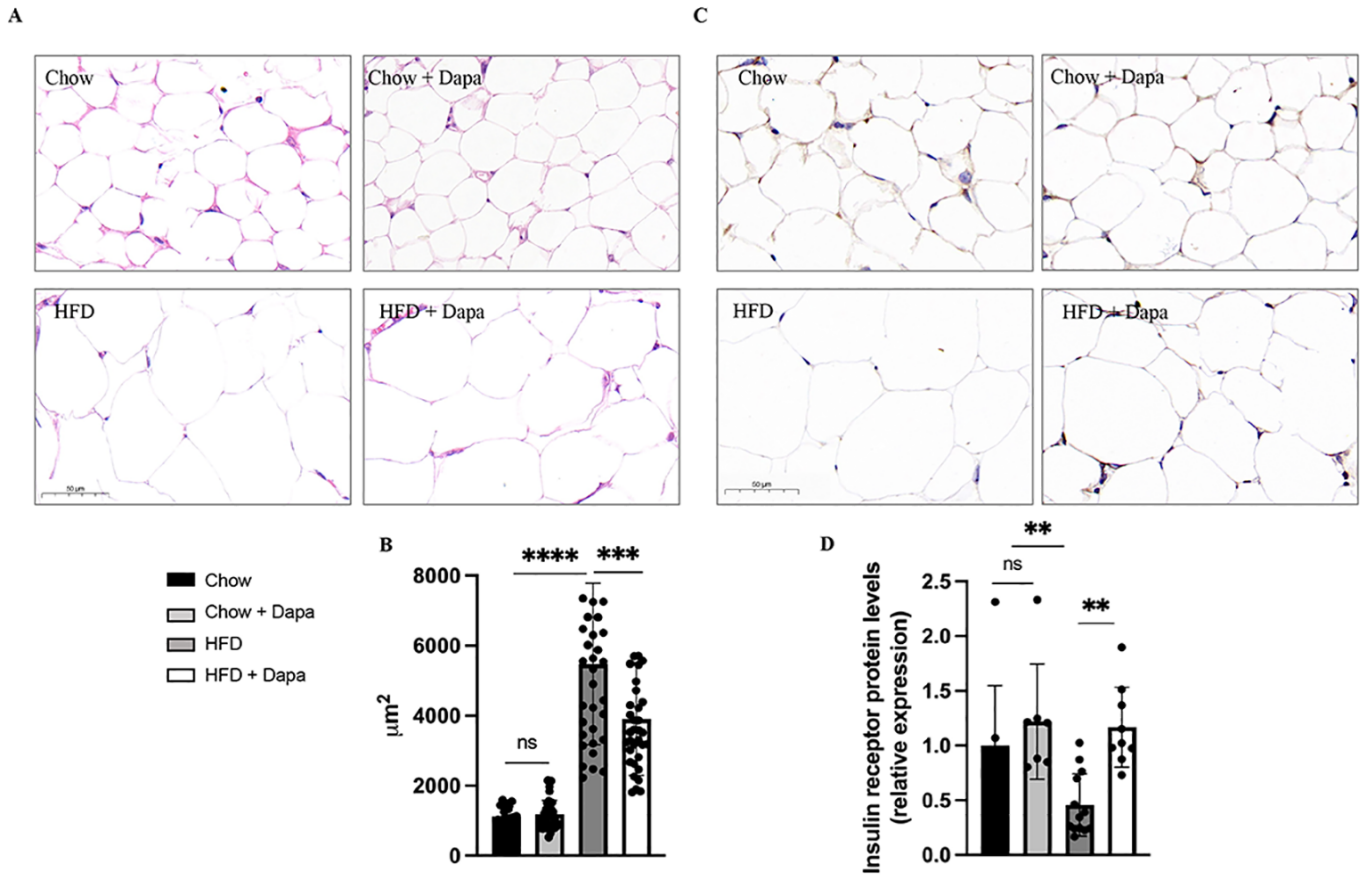
HE staining and immunohistochemistry of IR performed on histological sections of the peri-ovarian adipose tissue. **(A)** The representative HE staining images of mice; **(B)** Analysis of adipocyte area; **(C)** The representative immunohistochemical staining images of IR; **(D)** Quantitative analysis of IR. ^**^ *P* < 0.01, ^***^ *P* < 0.001, ^****^ *P* < 0.0001, HFD or HFD + Dapa vs Chow or Chow + Dapa. n = 4/group. The data was analyzed using ANOVA with Tukey’s test. Data are showed as means SD. Bar = 50um. ns, no significance. HE, Hematoxylin-eosin; IR, Insulin receptor.

Additionally, HE stained histological sections of periovarian adipose tissue from HFD-fed mice showed hypertrophy compared to chow-fed mice; However, this was improved after dapagliflozin treatment (**Figures 4A, B**). Furthermore, insulin receptor (IR) expression in periovarian adipose tissue was reduced in HFD-fed mice but significantly ameliorated after dapagliflozin treatment (**Figures 4C, D**).

### Glucose metabolism and insulin resistance

**Figure 5:**
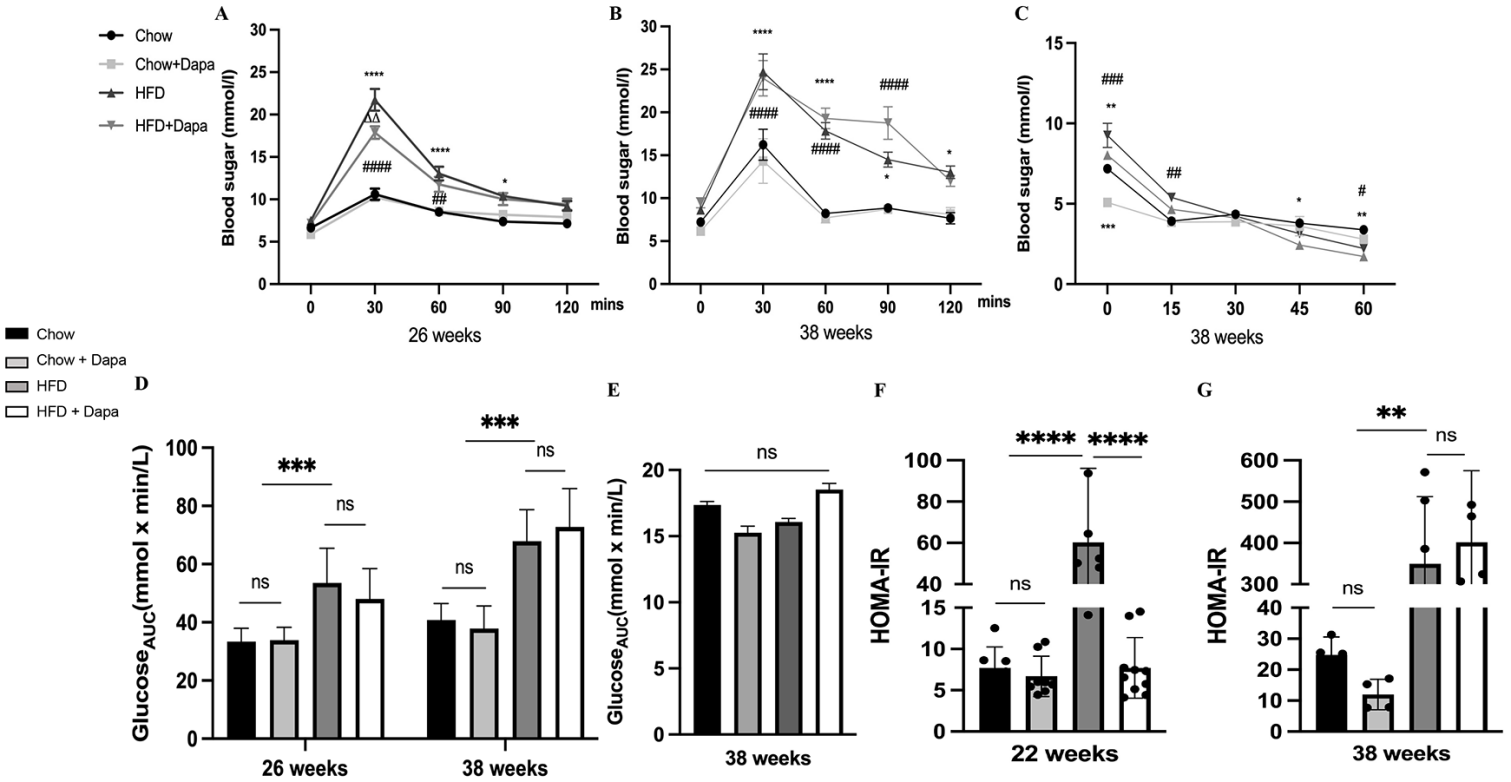
Consumption of HFD disrupted glucose tolerance, and leading to severe insulin resistance. **(A)** IPGTT was performed at 26 weeks of age; **(B)** IPGTT was performed at 38 weeks of age; **(C)** IPITT was performed at 38 weeks of age; **(D)** The AUC of IPGTT; **(E)** The AUC of IPITT; **(F)** The HOMA-IR index of mice at 26 weeks of age; **(G)** The HOMA-IR index of mice at 38 weeks of age. 26 weeks, n = 8-12/group, 38 weeks, n = 4-6/group. The data was analyzed using ANOVA with Tukey’s test. Data are showed as means SD. ^*^ *P* < 0.05, ^**^ *P* < 0.01, ^***^ *P* < 0.001, ^****^ *P* < 0.0001, HFD vs Chow or Chow + Dapa or HFD + Dapa; ^#^ *P* < 0.05, ^##^ *P* < 0.01, ^###^ *P* < 0.001, ^####^ *P* < 0.0001, HFD + Dapa vs Chow or Chow + Dapa; ^△△^ *P* < 0.01, HFD vs HFD + Dapa. ns, no significance. IPGTT, Intraperitoneal glucose tolerance tests; IPITT, Insulin tolerance tests; AUC, Area under the curve.

IPGTT were performed at 26-week and 38-week age, while IPITT was only conducted at 38 weeks. HFD feeding resulted in increased blood glucose levels at 30, 60, and 90 minutes after glucose injection compared to the chow and Chow + Dapa groups. However, the increase at 30 minutes was prevented in the HFD group with dapagliflozin treatment for 26 weeks (**Figure 5A**). The HFD group with dapagliflozin treatment for 38 weeks had similar glucose tolerance to those fed with the HFD (**Figure 5B**). Consumption of HFD increased the area under the curve (AUC) compared to the chow diet, which was not ameliorated by dapagliflozin (**Figure 5D**). To assess the effect of dapagliflozin on insulin sensitivity, mice underwent IPITT at the end of the experiment. Following a 6-hour fast, glucose levels in HFD-fed mice were significantly higher than those in chow-fed mice. Blood sugar levels were also significantly higher in HFD-fed dapagliflozin-treated mice than in chow-fed mice at 15 minutes after insulin injection. At both 45- and 60- minute post-injection, HFD-fed mice showed hypoglycemia, their blood sugar levels were markedly lower compared to those of the chow-fed groups (**Figure 5C**). The AUC did not differ significantly among all four groups (**Figure 5E**). At 22 weeks, mice fed a HFD showed a higher index of HOMA-IR compared to the other three groups (**Figure 5F**). However, the HOMA-IR index of HFD-fed mice was similar to that of HFD-fed mice treated with dapagliflozin (**Figure 5G**).

These findings demonstrate that the consumption of HFD disrupts glucose tolerance, leading to severe insulin resistance. However, it was observed that dapagliflozin could alleviate this damage at an early stage.

### Insulin, leptin, and inflammatory factors

**Figure 6:**
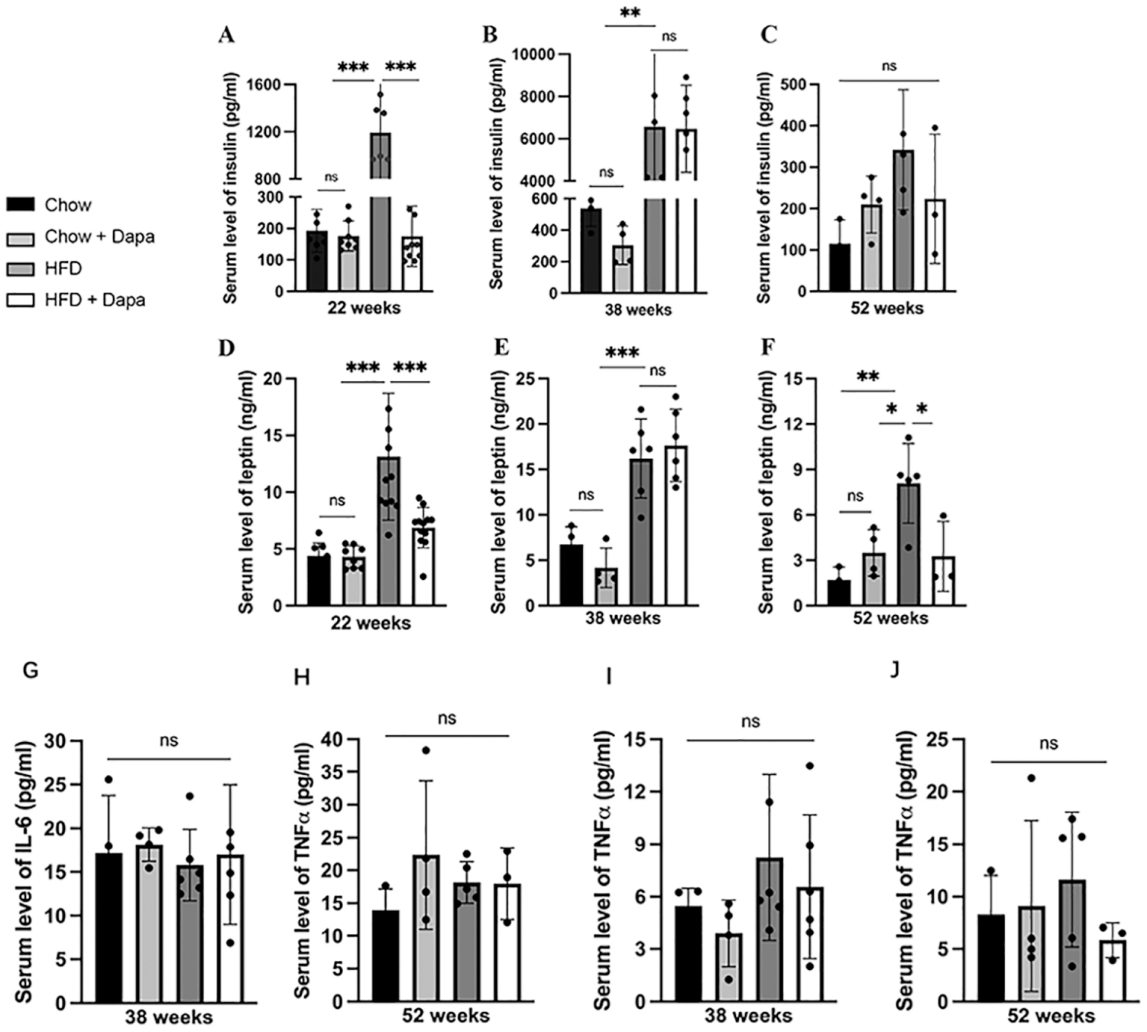
The serum levels of insulin, leptin, and inflammatory factors. Insulin: **(A)** age of 26 weeks; **(B)** age of 38 weeks; **(C)** age of 52 weeks; Leptin: **(D)** age of 26 weeks; **(E)** age of 38 weeks; **(F)** age of 52 weeks; IL-6: **(G)** age of 38 weeks; **(H)** age of 52 weeks; TNFα **(I)** age of 38 weeks; **(J)** age of 52 weeks. 26 weeks, n = 8-12/group, 38 weeks, n = 4-6/group, 52 weeks, n = 3-5/group. The data was analyzed using ANOVA with Tukey’s test. Data are showed as means SD. ^*^ *P* < 0.05, ^**^ *P* < 0.01, ^***^ *P* < 0.001, HFD vs Chow or Chow + Dapa or HFD + Dapa. ns, no significance.

Insulin and leptin are associated with the development of obesity. After 16 weeks of HFD feeding, we found significant increases in serum insulin and leptin levels, which were effectively mitigated by dapagliflozin treatment. In 22-week-old HFD-induced obese mice, circulating insulin levels exhibited an almost 5-fold rise, while circulating leptin levels showed a nearly to 1.5-fold rise. Dapagliflozin significantly improved insulin and leptin resistance in HFD-fed mice at this age, as shown in **Figures 6A, D**, however, no such improvement was noted in HFD-fed mice at the age of 38 weeks (**Figures 6B, E**). At the end of the experiment, serum insulin and leptin levels were markedly reduced correlating with body weight loss in both HFD feeding groups. Mice fed with HFD displayed significantly elevated leptin levels compared to the other three groups (**Figures 6C, F**).

Surprisingly, no significant differences were observed in serum IL-6 and TNFα among the four groups of mice at both 38 weeks and 52 weeks of age (**Figures 6G-J**).

### Changes in estrous cycle

**Figure 7:**
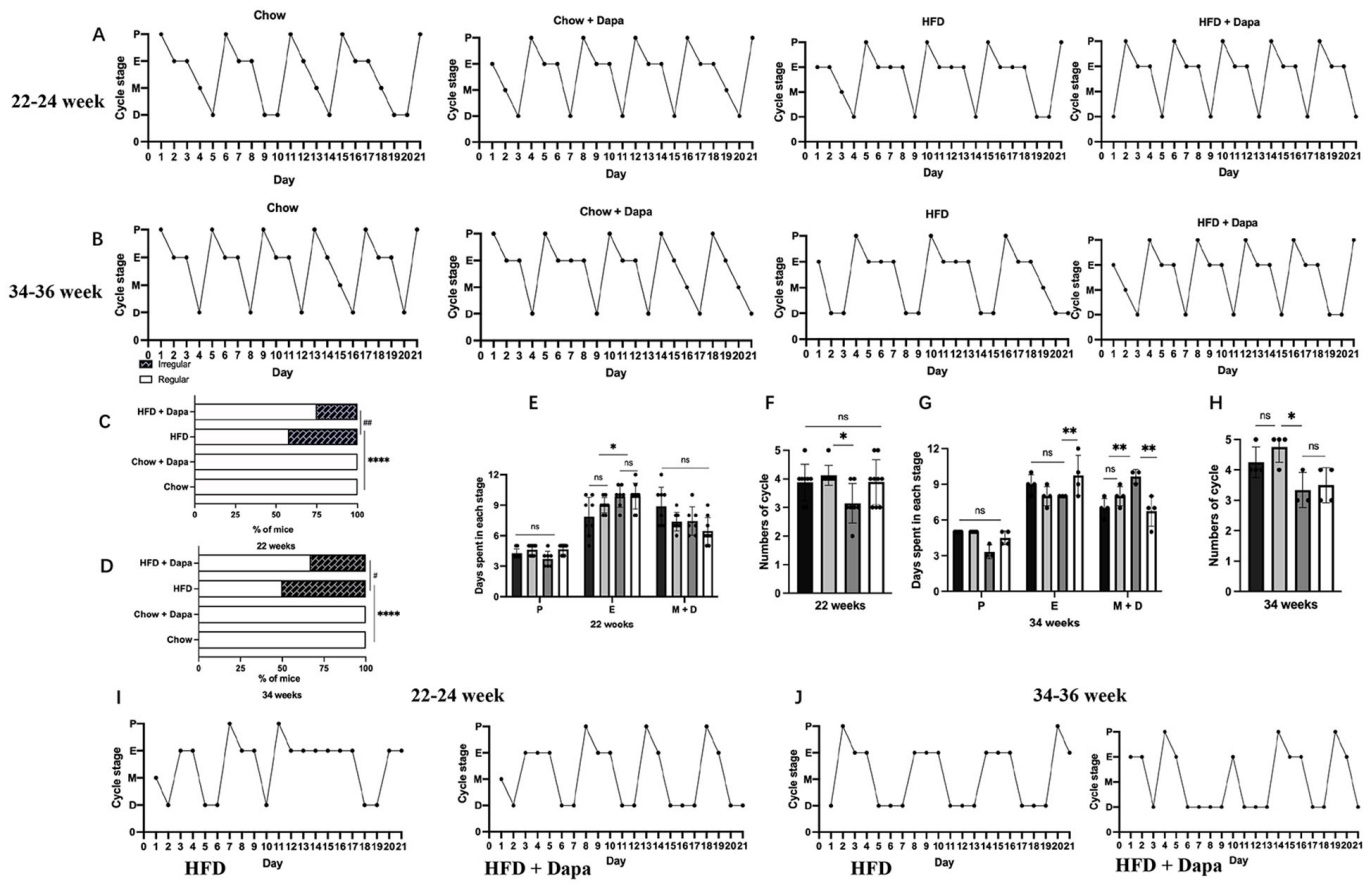
Changes in estrous cycle: **(A)** Representative regular estrous cycles of mice at 22-24 weeks of age; **(B)** Representative regular estrous cycles of mice at 34-36 weeks of age; **(C)** Percentage of mice with irregular estrous cycles at 22-24 weeks of age; **(D)** Percentage of mice with irregular estrous cycles at 34-36 weeks of age; 22-24 weeks: **(E)** Days spent in each stage over a 21-day period; **(F)** Numbers of cycle; 34-36 weeks: **(G)** Days spent in each stage over a 21-day period; **(H)** Number of cycles; **(I)** Representative irregular estrous cycles of mice at 22-24 weeks of age; **(J)** Representative irregular estrous cycles of mice at 34-36 weeks of age. 22-24 weeks, n = 8-12/group, 34-36 weeks, n = 4-6/group. The data was analyzed using ANOVA with Tukey’s test. A Chi-square test was used for non-parametric data. Data are showed as means SD. ^*^ *P* < 0.05, ^**^ *P* < 0.01, ^****^ *P* < 0.0001, HFD vs Chow or Chow + Dapa or HFD + Dapa; ^#^ *P* < 0.05, ^##^ *P* < 0.01, HFD + Dapa vs HFD. ns, no significance.

Vaginal cytology was performed continuously over a 28- day in mice aged 21 to 24 weeks and 33 to 36 weeks. The results of vaginal smears in the first week were not analyzed. All chow-fed mice showed regular estrous cycles. **Figures 7A, B** showed representative examples of these regular estrous cycles in mice. In the group of HFD-fed mice aged 22 to 24 weeks old, 42% (5/12) showed impaired estrous cycles, however, dapagliflozin treatment improved this condition, reducing the incidence of impairment to 25.5% (3/12) (**Figure 7C**). HFD-fed mice with regular cycles had fewer proestrus days compared to chow-fed mice, although dapagliflozin treatment ameliorated this issue without reaching statistical significance. Both HFD feeding and dapagliflozin treatment resulted in an extension of the estrus phase when compared to chow-fed mice (**Figure 7E**).

Furthermore, the HFD group experienced longer estrus cycle and fewer total cycles than those in the chow group, with significant differences observed only between the HFD group and those from the chow group receiving dapagliflozin (**Figure 7F**). At ages ranging from 34 to 36 weeks, half (50%, or 3/6) of HFD-fed mice showed impaired estrous cycles, conversely, only one-quarter (25%, or 2/6) of HFD-fed mice treated with dapagliflozin exhibited irregular cycles patters (**Figure 7D**). Long-term exposure to a HFD resulted in extended diestrus phase for these mice relative to other groups, additionally, those receiving dapagliflozin showed an extended duration within their estrous stage compared with other groups (**Figure 7G**). Moreover, over a span of 21 days, HFD-fed mice had fewer complete reproductive cycles than any other groups (**Figure 7H**). **Figures 7I, J** showed representative irregular estrous cycles observed among both HFD-fed mice as well as those treated with dapagliflozin at ages spanning from either 22-24 or 34-36 weeks old.

### Changes in sex hormones

The serum levels of LH and E2 were evaluated during the proestrus stage to assess the impact of dapagliflozin on ovulation. HFD-fed mice demonstrated a 1.5-fold increase in LH release compared to those fed chow mice. Although there was a tendency for decreased serum LH levels in HFD-fed mice treated with dapagliflozin, this difference did not reach statistical significance. Furthermore, E2 levels in HFD-fed mice were significantly elevated compared to those in chow-fed groups during both the proestrus and estrus stages, demonstrating that dapagliflozin had no discernible effect on estrogen secretion. These findings suggest that dapagliflozin may ameliorate ovulatory disorders by attenuating LH secretion (**Table 1**).

**Table 1:** Effects of HFD and dapagliflozin treatment on hormonal profile.

Group	Chow		HFD	
Dapagliflozin administration	(-)	(+)	(-)	(+)
28 weeks				
Number of sample (n)	8	8	10	10
LH (Proestrus stage, ng/ml)	6.46±0.65	5.56±0.48	9.07 ±.88^**^	7.65 ± 0.36
E2 (Proestrus stage, pg/ml)	549.70 ±40.27	571.34 ±32.30	866.81 ±66.04^***^	802.69 ±20.08^△△^
E2 (Estrus stage, pg/ml)	774.90 ±76.10	777.59 ±37.69	1010.94 ± 60.75^*^	1066.41 ± 45.98^△△^
38 weeks				
Number of sample (n)	4	4	6	6
LH (Diestrus stage, ng/ml)	6.11±0.36	4.81 ±0.68	8.94 ±1.83^*^	5.87 ±1.04^#^
E2 (Diestrus stage, pg/ml)	484.52 ±46.18	311.09 ±37.57	481.97 ±54.25	370.18 ±29.48
P (Diestrus stage, ng/ml)	5.13 ±0.69	3.93 ±0.17	1.72 ±0.58	5.54 ±1.58^##^
T (Diestrus stage, pg/ml)	45.09 ±5.80	32.40 ±5.72	95.21 ±6.19^**^	65.37 ±9.80^#^
52 weeks				
Number of sample (n)	4	4	5	3
LH (Diestrus stage, ng/ml)	11.83 ±0.77	10.56 ±0.48	6.52 ±0.34^**^	5.91 ±1.00^△△^
E2 (Diestrus stage, pg/ml)	750.14 ±115.33	760.08 ±148.60	380.13 ±30.40^**^	221.22 ±17.50^△△^
P (Diestrus stage, ng/ml)	8.40 ±2.60	10.93 ±1.11	7.09 ±1.25	3.89 ±0.70^#^
T (Diestrus stage, pg/ml)	198.96 ±67.33	146.73 ±33.03	97.99 ±26.30	45.56 ±5.80^△△^

Chow and HFD groups had treatment with dapagliflozin (Dapagliflozin administration +), had no treatment with dapagliflozin (Dapagliflozin administration -). Values are mean ± SD. ^*^ *P <* 0.05, ^**^P < 0.01, ^***^P < 0.001, compared with untreated Chow group or treated Chow group; ^#^ *P <* 0.05, ^##^ *P <* 0.01, compared with untreated HFD group; ^△^ *P <* 0.05, ^△△^ *P <* 0.01, compared with t untreated Chow group. E2: estradiol2; LH: luteinizing hormone; P: progesterone; T: testosterone.

We investigated sex hormone levels in 38-week-old mice during the diestrus stage. HFD-fed mice exhibited elevated LH levels compared to other groups, while E2 levels remained consistent across all groups and were lower than those observed during the proestrus and estrus stages. Additionally, HFD-fed mice demonstrated reduced P levels relative to those receiving dapagliflozin treatment, alongside increased T level when compared to the other group of mice (**Table 1**). These findings suggest that dapagliflozin may enhance the balance of sex hormone secretion, with the exception of E2 release.

Although the sample size for 52-week-old mice was limited, the HFD-fed mice showed significantly lower levels of LH and E2 compared to their chow-fed mice. These findings suggest that prolonged consumption of a HFD leads to a reduction in E2 release, which subsequently results in decreased LH secretion (**Table 1**). It is important to note that only three mice were included in the HFD-fed group receiving dapagliflozin treatment, therefore, the results regarding hormone secretion may not be entirely reliable.

### Pregnancy and perinatal outcomes following HFD and dapagliflozin treatment

To further evaluate the impact of a HFD and dapagliflozin on pregnancy outcomes, we housed two female and one male mice in a single cage. The pregnant females delivered naturally, and we recorded both the number of pups born and their birth weight (BW). After six weeks, we found that all chow-fed mice (100%) successfully delivered pups. In contrast, among the HFD group, three out of six mice (50%) achieved pregnancy, only two of these mice gave birth (33.3%); while, one mouse experienced an abortion followed by death. Dapagliflozin treatment improved the pregnancy rate in HFD-fed mice to some extent, with five out of six becoming pregnant (83.33%), unfortunately, three out of these five pregnant mice died during childbirth. Chow-fed mice produced 16 pups with an average BW of 1.43, whereas chow-fed mice treated with dapagliflozin had 18 pups with an average BW of 1.39. Conversely, HFD-fed mothers gave birth to 3 pups averaging a BW of 2.33, those treated with dapagliflozin experienced stillbirths weighing an average of 1.90 alongside live births averaging a weight of 1.48. Notably, the BW of pups from HFD maternal mice was heavier than that from chow maternal mice and was similar to that for stillbirths from HFD-fed maternal mice previously treated with dapagliflozin (**Table 2**).

**Table 2:** Maternal outcome and offspring (one male and two female mice in the cage for 6 weeks or 10 weeks).

Group	Offspring number	Pups Birth weight (g)	Means ± SEM
Chow			
1	5	1.5, 1.6, 1.8, 1.1, 1.1	
2	2	1.4, 1.5	
3	6	1.5, 1.6, 1.3, 1.1, 1.2, 1.4	
4	3	1.3, 1.7, 1.8	1.43 ±0.06
Chow + Dapa			
1	7	1.5, 1.4, 1.3, 1.1, 1.2, 1.4,1.5	
2	2	1.4, 1.5	
3	7	1.5, 1.6, 1.3, 1.1, 1.2, 1.4,1.4	
4	2	1.6, 1.7	1.39 ±0.04
HFD			
1	Miscarry (dead)		
2	1	2.4 (Stillbirth)	
3	2	2.5, 2.1 (Stillbirth)	
4			
5			
6			2.33 ±0.12^****^
HFD + Dapa			
1	1 (dead)	1.9 (Stillbirth)	Live birth
2	1	1.45	1.48 ± 0.17^△△△^
**3**	2	1.2, 1.8	
**4**	3 (dead)	1.8, 1.7, 2.2 (Stillbirth)	
**5**			Stillbirth
6	1 (dead)	1.9 (Stillbirth)	1.90 ±0.08 ^###^

Pregnancy and perinatal outcome. Values are mean ± SD. ****P < 0.0001, compared with untreated Chow group or treated Chow group; ^###^ *P <* 0.001, compared with untreated Chow group or treated Chow group; ^△△△^ *P <* 0.001, compared with untreated HFD group.

The study demonstrated that a HFD resulted in decreased fertility among female mice. Dapagliflozin was found to ameliorate this fertility impairment caused by HFD, however, it also led to increased mortality rates during childbirth in oversized offspring.

### Effects of HFD and dapagliflozin on metabolism in adult offspring

After observing the effects of a HFD and dapagliflozin on pregnancy and perinatal outcomes in female mice, we housed both the chow group and HFD group females with male mice for a duration of 4 weeks. The control group females gave birth to 4 males and 5 females, while those in the HFD group produced 4 males and 4 females. In the subsequent phase of our study, we measured the weight and fasting blood sugar levels of adult offspring. Notably, the fasting blood sugar levels of male offspring from maternal mice in both chow-fed or HFD-fed groups were significantly higher than those of their female offspring (**Table 3**). Interestingly, there was no significant difference observed in body weight between female offspring born to chow-fed mothers versus those born to HFD-fed mothers. However, at 16 weeks old, male offspring from HFD-fed maternal mice exhibited significantly greater body weight compared to their counterparts from chow-fed maternal mice. Furthermore, male pups from HFD-fed maternal mice demonstrated increased subperitoneal fat relative to those from the chow group (**Table 3**).

**Table 3:** Fasting blood sugar and weight of offspring in chow group and HFD group.

Group	Chow		HFD	
	Male(n=4)	Female (n=5)	Male(n=4)	Female(n=4)
Blood sugar	6.55 ±0.33^#^	5.14 ±0.26	6.78 ±0.28^#^	5.45 ±0.29
Weight				
10 weeks	24.87 ±0.74	19.74 ±0.54	25.51 ±0.72	19.27 ±0.42
16 weeks	25.26 ±0.67	21.36 ±0.31	28.28 ±0.81^*^	21.12 ±0.57
Subcutaneous fat	0.17 ±0.01	0.19 ±0.01	0.22 ±0.01	0.23 ±0.02
Subperitoneal fat	0.37 ±0.03	0.25 ±0.03	0.45 ±0.02^*^	0.30 ±0.04

Fasting blood sugar and weight of offspring. ^#^ *P <* 0.05, compared with chow or HFD group maternal mice; ^*^ *P <* 0.05, compared with male offspring from chow maternal mice.

The study indicates that the metabolic status of maternal mice exerts a more influence on the metabolism of their male offspring.

### Number of corpora lutea in the ovary

**Figure 8:**
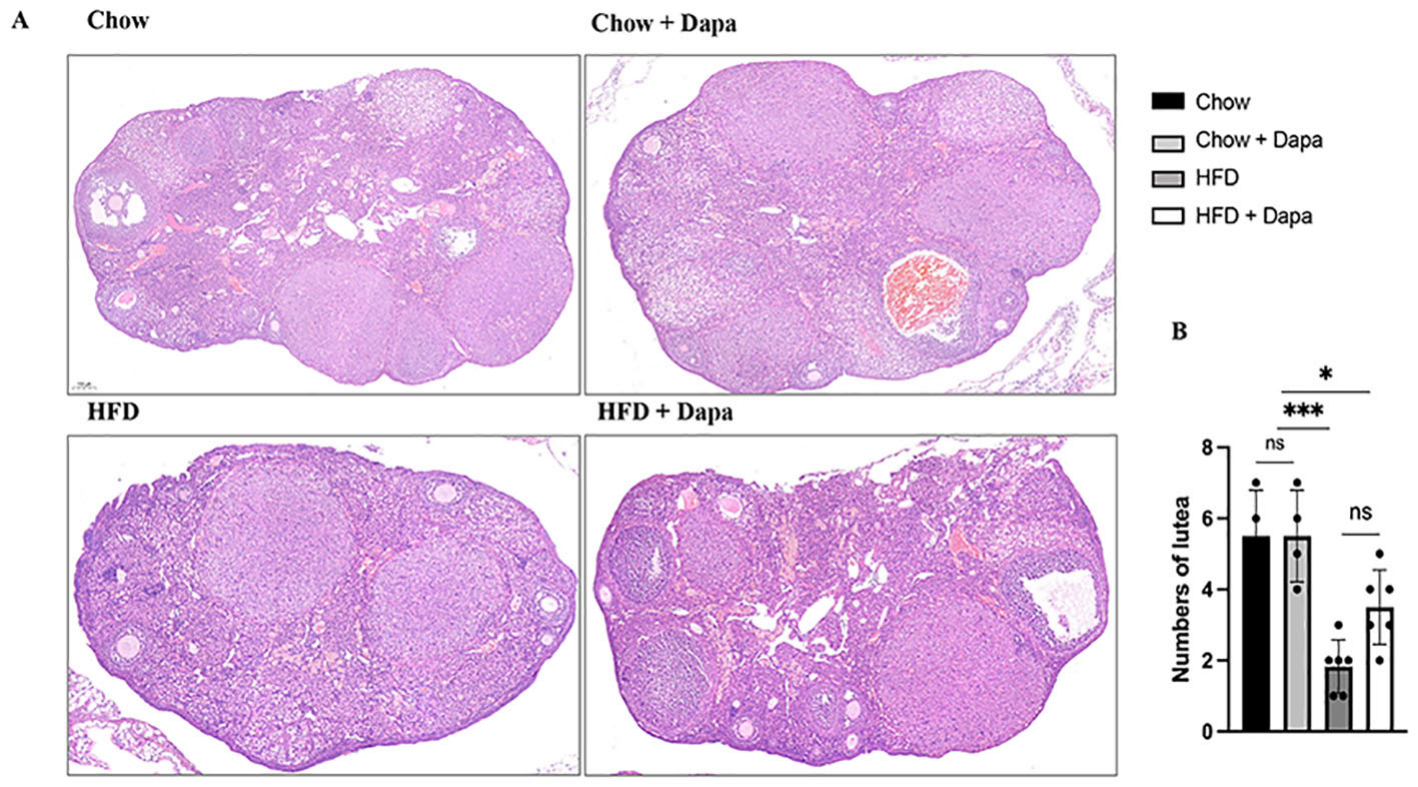
Numbers of lutea. **(A)** The representative HE staining images of ovary; **(B)** Quantitative analysis of numbers of lutea, n = 4/group. The data was analyzed using ANOVA with Tukey’s test. Data are showed as means SD. ^*^P < 0.05, HFD + Dapa vs Chow or Chow + Dapa; ^***^ *P* < 0.001, HFD vs Chow or Chow + Dapa. ns, no significance. Bar = 100um.

The number of corpora lutea was quantified as an indicator of ovulation in the ovaries of 38-week-old mice during diestrus. Histological sections stained with HE demonstrated that HFD-fed mice exhibited a reduced number of corpora lutea compared to chow-fed mice. In contrast, HFD-fed mice treated with dapagliflozin showed an intermediate count (**Figures 8A, B**). This result aligns with the observed decrease in proestrus days and lower litter size among HFD-induced obese females.

### Insulin receptor expression in the pituitary gland

**Figure 9:**
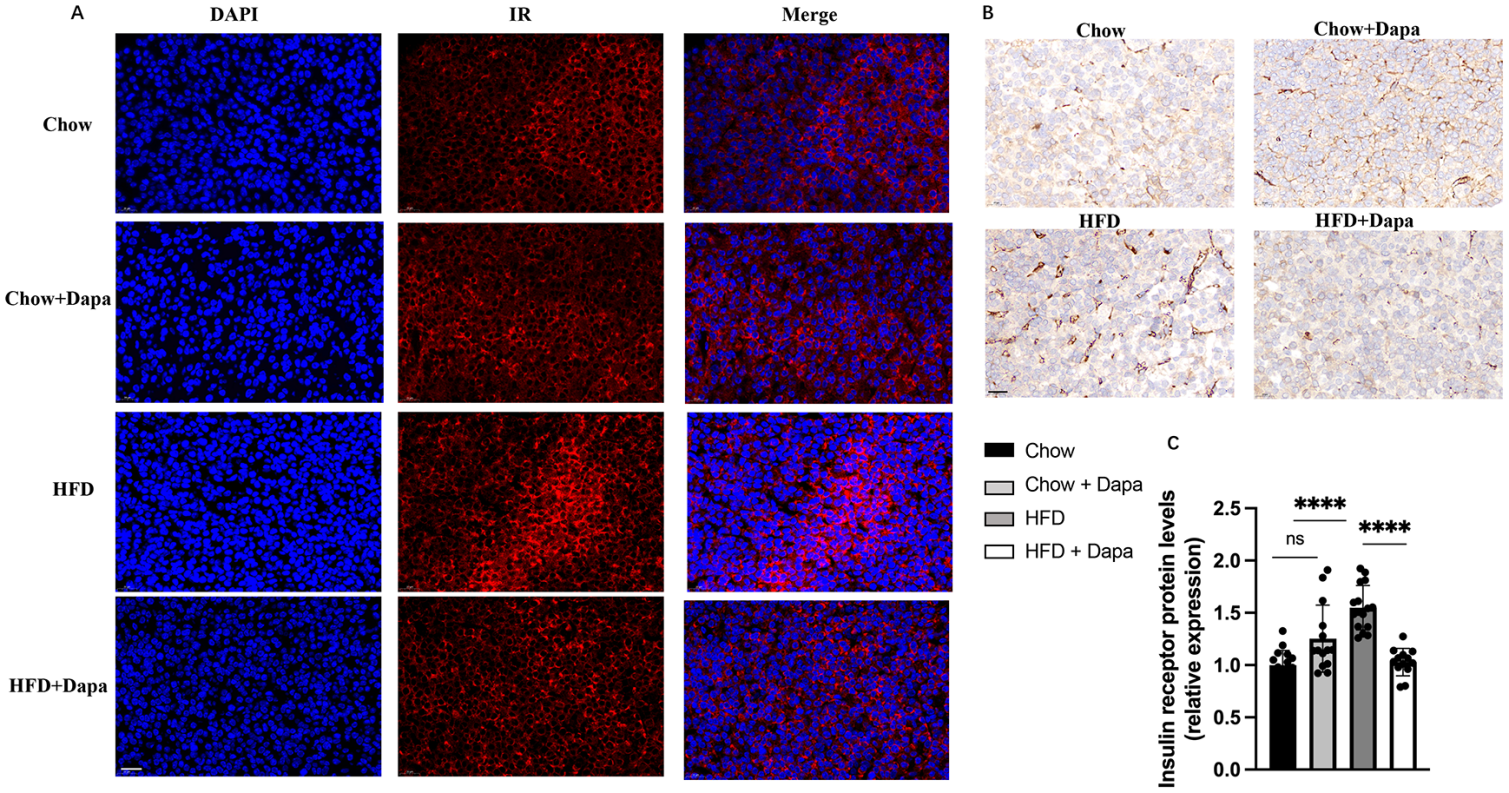
IR expression on pituitary. **(A)** Representative immunofluorescence images of IR labeling (red), counterstained with DAPI; **(B)** Representative immunohistochemistry stained with IR images; **(C)** Quantitative analysis of IR; n = 4/group. The data was analyzed using ANOVA with Tukey’s test. Data are showed as means SD. ^****^ *P* < 0.0001, HFD vs Chow or Chow + Dapa or HFD + Dapa; ns, no significance. Scale bar = 20 μm.

IR expression in pituitary gland was evaluated to elucidate its role in infertility associated with diet-induced obesity. Immunofluorescence and immunohistochemistry staining confirmed a high level of IR expression within the pituitary gland, with significantly elevated levels observed in HFD mice compared to those on chow diet (**Figures 9A, B**). However, treatment with dapagliflozin in HFD mice resulted in a significant decrease in IR expression, which contrasts with previous finding regarding hypothalamic IR expression (**Figure 9C**).

## Discussion

In previous research, we discovered that inhibiting the expression of SGLT2 markedly ameliorated hypothalamic inflammation and the proinflammatory polarization of microglia, thereby enhancing ovulation disorders (5). To minimize the impact of macrophages from peripheral adipose tissue infiltrating the hypothalamus and inducing inflammation, we concurrently administered a HFD and dapagliflozin to investigate the effects of the SGLT2i on central inflammation. In addition, we have previously examined the influence of SGLT2i on metabolism and reproduction in obese MC4RKO mice, exploring the potential underlying mechanisms (6). Furthermore, mice with ovulatory disorders induced by a HFD serve as ideal models for investigating ovulatory infertility associated with elevated HFD intake (18, 19).

The current findings indicate that the early administration of dapagliflozin can mitigate visceral fat accumulation, weight gain, glucose intolerance, insulin resistance, and ovulatory dysfunction induced by HFD. However, it was observed that the beneficial effects of dapagliflozin on HFD-induced metabolic disorders gradually disappeared over time. At 26 weeks of age, no significant differences were observed in the fasting blood glucose levels among the four groups of mice, contrasting with our prior findings where HFD-fed mice exhibited higher fasting blood glucose compared to the control group. This discrepancy might be related to the sample size or the diminished impact of HFD on blood glucose in female mice, but the outcomes of the glucose tolerance tests remained consistent across both experiments (5). Nevertheless, in mice fed a HFD, dapagliflozin’s improvement in the estrous cycle, adipocyte volume, and tissue insulin receptor expression were sustained. Dapagliflozin is a highly selective SGLT-2i known to lower blood sugar by inhibiting renal tubular absorption of glucose (20, 21). These findings indicate that the positive effects of dapagliflozin on reproductive function in HFD-fed mice are not exclusively attributed to its role in glucose excretion for metabolic improvement; other pathophysiological mechanisms are likely to be involved. Our previous study has demonstrated that SGLT2 is significantly expressed in microglia and hypothalamus. These finding suggests that dapagliflozin can effectively inhibit microglial proinflammatory activation, prevent HFD-induced hypothalamic inflammation and weight gain, and ameliorate ovulation disorders (5).

So, we speculated that: a) HFD feeding induces proinflammatory activation of microglia, which lead to hypothalamic inflammation, obesity, and ovulation disorders. Treatment with dapagliflozin may mitigate hypothalamic inflammation by inhibiting microglia proinflammatory activation, thereby improving both obesity and ovulation disorders. b) Prolonged HFD consumption and subsequent weight gain result in the infiltration of peripherally derived macrophages across the blood-brain barrier into the hypothalamus, exacerbating inflammation. Dapagliflozin treatment is ineffective in inhibiting the release of inflammatory factors from these peripherally derived macrophages or ameliorating hypothalamic inflammation; consequently, it cannot prevent HFD-induced weight gain or improve metabolic abnormalities associated with obesity. In future research, we will focus on investigating the expression of SGLT2 in hypothalamic microglia and its functional implication, as well as its impact on GnRH neuron functionality. Additionally, we will explore how SGLT2 inhibition influences adipocyte volume and insulin receptor expression along with elucidating the underlying mechanisms involved.

At age of 22 to 24 weeks, compared to the chow-fed group, HFD-fed mice with a regular estrous cycle exhibited a prolonged estrous cycle and an extended duration of the estrous period. Dapagliflozin treatment effectively restored the estrous cycle in HFD-fed mice; however, the duration of the estrous period remained unchanged. We hypothesize that the HFD facilitated follicular development, resulting in an extended estrous period and elevated E2 levels in these mice.

In the present study, mice fed with HFD exhibited elevated serum LH levels during proestrus and diestrus, increased serum E2 levels during proestrus and estrus, higher serum T levels and lower serum P levels during diestrus, as well as irregular estrous cycle patterns and reduced ovulation. The hormonal profiles and ovulatory disorders observed in HFD-fed mice exhibit similarities to those found in obese women diagnosed with polycystic ovary syndrome (PCOS) (22, 23). Dapagliflozin treatment significantly reduced serum LH secretion during the proestrus stage in HFD-fed mice without altering serum E2 levels. These findings suggest that the ameliorative effects of dapagliflozin on HFD-induced ovulation disorders may be linked to the normalization of LH secretion. The elevated E2 levels observed during the proestrus and estrus stages in HFD-fed mice are likely correlated with follicular development. In our previous research, we observed a greater number of preovulatory follicles in the ovaries of both HFD-fed mice and MC4R KO obese mice during estrus stage compared to the control group. This finding aligns with an extended duration of the estrus stage observed within both HFD groups. Furthermore, HFD-fed mice exhibited significantly lower serum P levels during the diestrus stage when compared to chow-fed mice, which was associated with ovulation disorders as well as a reduced luteal count among HFD-fed mice.

In addition, we found that 52- week-old HFD-fed mice displayed significantly decreased serum LH and E2 levels during diestrus stage (after 18 weeks of discontinuing HFD feeding). This suggests that the long-term hypogonadotropic hypogonadism induced by HFD is mediated through suppression of GnRH. This finding aligns with our previous study, which demonstrated a reduction in both neuronal number and protein expression of GnRH and Kisspeptin in the hypothalamus of HFD-induced obese mice. This finding requires further validation through a large-scale sample study.

The previous study has reported that insulin signaling within the pituitary plays an important role in ovulation disorders related to obesity induced by HFD. The knockout of IR in the pituitary has been shown to alleviate reproductive impairment (24, 25). Furthermore, it remains unclear whether SGLT2 is expressed in the pituitary gland. In this study, mice subjected to HFD displayed impaired reproductive function alongside an upregulation of IR expression in the pituitary. However, treatment with dapagliflozin in HFD-fed mice resulted in improved ovulatory function and a significant downregulation of pituitary IR, despite persistent hyperinsulinemia, hyperleptinemia, and obesity. These results further support the notion that insulin signaling within the pituitary, rather than insulin resistance, is fundamental to regulating of LH secretion associated with HFD-induced obesity. Additionally, our study revealed no evidence of SGLT2 expression in pituitary cells by immunofluorescence analysis (data not shown). This suggests that alternative compensatory mechanisms may contribute to the improvement of reproductive function against HFD-induced upregulation of pituitary IR. These findings warrant confirmation and further elucidation through additional studies.

Previous clinical studies have shown that treatment with SGLT-2i in individuals with type 2 diabetes can lead to an increase in endogenous glucose production (26–29). Our research has also revealed that dapagliflozin treatment in female mice exerts a preventive effect against hypoglycemia induced by insulin. Additionally, it has been reported that chronic administration of SGLT-2i may induce metabolic reprogramming, resulting in decreased glucose disposal and enhanced lipid utilization (30). However, previous studies on the effects of SGLT-2i on energy homeostasis, ectopic fat accumulation, and obesity-induced insulin resistance and inflammation were exclusively conducted using male rodent models (31). The present study shows that 16 weeks of dapagliflozin treatment following 16 weeks of HFD feeding attenuates weight gain, fat accumulation, insulin resistance, and leptin resistance in female mice. Notably, these beneficial effects were not observed after 32 weeks of HFD feeding. Furthermore, our findings indicate that dapagliflozin does not attenuate obesity-related inflammation in 32-week-old HFD-fed mice. A significant finding from this study is that dapagliflozin significantly suppressed the increases in peri-ovarian adipocyte sizes induced by HFD while concurrently decreasing the expression of IR within adipocytes. This data is consistent with previous studies indicating that SGLT-2i promote lipolysis within the adipose tissue of mice (31–33).

To the best of our understanding regarding the effects of HFD and dapagliflozin on mouse fertility, this study represents the first evidence that dapagliflozin treatment can improve the pregnancy rate in HFD-induced obese mice while simultaneously increasing maternal mortality rates. As anticipated, our findings indicate that dapagliflozin has the capacity to stimulate ovulation, as indicated by an increase in the number of corpora lutea observed in the ovaries. Taken together, these results suggest that dapagliflozin’s improvement of fertility in HFD-induced conditions is independent of obesity, insulin resistance, and inflammation. Conversely, previous clinical studies have established maternal obesity as a recognized risk factor for metabolic dysfunction in offspring, including those persisting into adulthood (4, 34, 35). Our research indicates that adult male offspring from HFD-induced obese mothers exhibited a greater propensity for weight gain and visceral fat accumulation compared to those from control diet mothers.

However, further research is needed to elucidate the role and central mechanism of microglia and its SGLT2 expression in hypothalamic inflammation, obesity, and ovulation disorders induced by HFD intake in mice. Additionally, the mechanism underlying the impact of a HFD on the processes of offspring from maternal mice require further investigation.

## Conclusion

In conclusion, the present study comprehensively observed the impacts of dapagliflozin-mediated SGLT2 inhibition on metabolic profile, sex hormones, ovulatory function, fertility, and offspring in female mice subjected to a long-term HFD. The HFD-induced obese C57BL/6J female mice showed metabolic and reproductive profiles similar to those observed in women with PCOS, including hyperinsulinemia, hyperleptinemia, hyperandrogenemia, and elevated LH levels. Treatment with dapagliflozin for 32 weeks following an initial 32-week period of HFD feeding did not attenuate obesity or insulin resistance, however, it did enhance reproductive function, decrease adipocyte hypertrophy, and improve LH release. These improvements were accompanied with a decrease in the expression of IRs within the pituitary. Furthermore, our previous study demonstrated that dapagliflozin alleviated hypothalamic inflammation and ameliorated neuronal damage induced by HFD. These results suggest a potential mechanism by which SGLT-2i may improve ovulatory dysfunction caused by HFD via attenuating HFD-induced hypothalamic inflammation and facilitating metabolic reprogramming. Overall, the current study highlights the potential clinical utility of SGLT-2i in the prevention and treating obesity-related infertility.

## Data Availability

The raw data supporting the conclusions of this article will be made available by the authors, without undue reservation.

## References

[1] Di Berardino C Peserico A Capacchietti G Zappacosta A Bernabo N Russo V. High-fat diet and female fertility across lifespan: A comparative lesson from mammal models. Nutrients. (2022) 14:1–32. doi: 10.3390/nu14204341 PMC9610022 36297035

[2] Hohos NM Elliott EM Giornazi A Silva E Rice JD Skaznik-Wikiel ME. High-fat diet induces an ovulatory defect associated with dysregulated endothelin-2 in mice. Reproduction. (2021) 161:307–17. doi: 10.1530/REP-20-0290 33428588

[3] Akhaphong B Gregg B Kumusoglu D Jo S Singer K Scheys J. Maternal high-fat diet during pre-conception and gestation predisposes adult female offspring to metabolic dysfunction in mice. Front Endocrinol (Lausanne). (2021) 12:780300. doi: 10.3389/fendo.2021.780300 35111136 PMC8801938

[4] Xu Y Yang D Wang L Krol E Mazidi M Li L. Maternal high fat diet in lactation impacts hypothalamic neurogenesis and neurotrophic development, leading to later life susceptibility to obesity in male but not female mice. Adv Sci (Weinh). (2023) 10:e2305472. doi: 10.1002/advs.202305472 37867217 PMC10724448

[5] Chen X Xiao Z Liu Q Luo D Cai Y Fan M. Dapagliflozin ameliorates ovulation disorders via attenuating activated microglia-mediated hypothalamic inflammation in HFD-fed mice. Neuroendocrinology. (2024) 114:331–47. doi: 10.1159/000535420 38147832

[6] Cui L Tan C Huang L Wang W Huang Z Geng F. Dapagliflozin partially restores reproductive function in MC4R KO obese female mice. J Endocrinol. (2022) 254:65–76. doi: 10.1530/JOE-21-0449 35612570

[7] Qiao J Wang Y Li X Jiang F Zhang Y Ma J. A Lancet Commission on 70 years of women's reproductive, maternal, newborn, child, and adolescent health in China. Lancet. (2021) 397:2497–536. doi: 10.1016/S0140-6736(20)32708-2 34043953

[8] Venkatesh SS Ferreira T Benonisdottir S Rahmioglu N Becker CM Granne I. Obesity and risk of female reproductive conditions: A Mendelian randomisation study. PloS Med. (2022) 19:e1003679. doi: 10.1371/journal.pmed.1003679 35104295 PMC8806071

[9] Carson SA Kallen AN. Diagnosis and management of infertility: A review. JAMA. (2021) 326:65–76. doi: 10.1001/jama.2021.4788 34228062 PMC9302705

[10] Liao B Qiao J Pang Y. Central regulation of PCOS: abnormal neuronal-reproductive-metabolic circuits in PCOS pathophysiology. Front Endocrinol (Lausanne). (2021) 12:667422. doi: 10.3389/fendo.2021.667422 34122341 PMC8194358

[11] Yang R Li Q Zhou Z Qian W Zhang J Wu Z. Changes in the prevalence of polycystic ovary syndrome in China over the past decade. Lancet Reg Health West Pac. (2022) 25:100494. doi: 10.1016/j.lanwpc.2022.100494 35669932 PMC9162959

[12] Mizgier M Jarzabek-Bielecka G Formanowicz D Jodlowska-Siewert E Mruczyk K Cisek-Wozniak A. Dietary and physical activity habits in adolescent girls with polycystic ovary syndrome (PCOS)-HAstudy. J Clin Med. (2021) 10:1–17. doi: 10.3390/jcm10163469 PMC8396824 34441766

[13] Javed Z Papageorgiou M Deshmukh H Rigby AS Qamar U Abbas J. Effects of empagliflozin on metabolic parameters in polycystic ovary syndrome: A randomized controlled study. Clin Endocrinol (Oxf). (2019) 90:805–13. doi: 10.1111/cen.13968 30866088

[14] Javed Z Papageorgiou M Madden LA Rigby AS Kilpatrick ES Atkin SL. The effects of empagliflozin vs metformin on endothelial microparticles in overweight/obese women with polycystic ovary syndrome. Endocr Connect. (2020) 9:563–69. doi: 10.1530/EC-20-0173 PMC7354739 32449697

[15] Tan S Ignatenko S Wagner F Dokras A Seufert J Zwanziger D. Licogliflozin versus placebo in women with polycystic ovary syndrome: A randomized, double-blind, phase 2 trial. Diabetes Obes Metab. (2021) 23:2595–99. doi: 10.1111/dom.14495 34263971

[16] Elkind-Hirsch KE Chappell N Seidemann E Storment J Bellanger D. Exenatide, dapagliflozin, or phentermine/topiramate differentially affect metabolic profiles in polycystic ovary syndrome. J Clin Endocrinol Metab. (2021) 106:3019–33. doi: 10.1210/clinem/dgab408 34097062

[17] Zhang J Xing C Cheng X He B. Canagliflozin combined with metformin versus metformin monotherapy for endocrine and metabolic profiles in overweight and obese women with polycystic ovary syndrome: A single-center, open-labeled prospective randomized controlled trial. Front Endocrinol (Lausanne). (2022) 13:1003238. doi: 10.3389/fendo.2022.1003238 36147577 PMC9486461

[18] Hohos NM Cho KJ Swindle DC Skaznik-Wikiel ME. High-fat diet exposure, regardless of induction of obesity, is associated with altered expression of genes critical to normal ovulatory function. Mol Cell Endocrinol. (2018) 470:199–207. doi: 10.1016/j.mce.2017.10.016 29097167

[19] Velazquez C Herrero Y Bianchi MS Cohen DJ Cuasnicu P Prost K. Beneficial effects of metformin on mice female fertility after a high-fat diet intake. Mol Cell Endocrinol. (2023) 575:111995. doi: 10.1016/j.mce.2023.111995 37364632

[20] Furtado RHM Bonaca MP Raz I Zelniker TA Mosenzon O Cahn A. Dapagliflozin and cardiovascular outcomes in patients with type 2 diabetes mellitus and previous myocardial infarction. Circulation. (2019) 139:2516–27. doi: 10.1161/CIRCULATIONAHA.119.039996 30882239

[21] Tamborlane WV Laffel LM Shehadeh N Isganaitis E Van Name M Ratnayake J. Efficacy and safety of dapagliflozin in children and young adults with type 2 diabetes: a prospective, multicentre, randomised, parallel group, phase 3 study. Lancet Diabetes Endocrinol. (2022) 10(5):341–50. doi: 10.1016/S2213-8587(22)00052-3 PMC10851108 35378069

[22] Podfigurna A Meczekalski B Petraglia F Luisi S. Clinical, hormonal and metabolic parameters in women with PCOS with different combined oral contraceptives (containing chlormadinone acetate versus drospirenone). J Endocrinol Invest. (2020) 43:483–92. doi: 10.1007/s40618-019-01133-3 PMC7067819 31654312

[23] Elkind-Hirsch KE Chappell N Shaler D Storment J Bellanger D. Liraglutide 3 mg on weight, body composition, and hormonal and metabolic parameters in women with obesity and polycystic ovary syndrome: a randomized placebo-controlled-phase 3 study. Fertil Steril. (2022) 118:371–81. doi: 10.1016/j.fertnstert.2022.04.027 35710599

[24] Brothers KJ Wu S DiVall SA Messmer MR Kahn CR Miller RS. Rescue of obesity-induced infertility in female mice due to a pituitary-specific knockout of the insulin receptor. Cell Metab. (2010) 12:295–305. doi: 10.1016/j.cmet.2010.06.010 20816095 PMC2935812

[25] Wu S Divall S Wondisford F Wolfe A. Reproductive tissues maintain insulin sensitivity in diet-induced obesity. Diabetes. (2012) 61:114–23. doi: 10.2337/db11-0956 PMC3237653 22076926

[26] Daniele G Solis-Herrera C Dardano A Mari A Tura A Giusti L. Increase in endogenous glucose production with SGLT2 inhibition is attenuated in individuals who underwent kidney transplantation and bilateral native nephrectomy. Diabetologia. (2020) 63:2423–33. doi: 10.1007/s00125-020-05254-w PMC7527374 32827269

[27] Abdelgani S Khattab A Adams J Abu-Farha M Daniele G Al-Mulla F. Distinct mechanisms responsible for the increase in glucose production and ketone formation caused by empagliflozin in T2DM patients. Diabetes Care. (2023) 46:978–84. doi: 10.2337/dc22-0885 PMC10154659 36857415

[28] Alatrach M Agyin C Solis-Herrera C Lavryneko O Adams J Gastaldelli A. Dapagliflozin impairs the suppression of endogenous glucose production in type 2 diabetes following oral glucose. Diabetes Care. (2022) 45:1372–80. doi: 10.2337/dc21-1798 PMC9531536 35235659

[29] Merovci A Solis-Herrera C Daniele G Eldor R Fiorentino TV Tripathy D. Dapagliflozin improves muscle insulin sensitivity but enhances endogenous glucose production. J Clin Invest. (2014) 124:509–14. doi: 10.1172/JCI70704 PMC3904617 24463448

[30] Xu L Nagata N Nagashimada M Zhuge F Ni Y Chen G. SGLT2 inhibition by empagliflozin promotes fat utilization and browning and attenuates inflammation and insulin resistance by polarizing M2 macrophages in diet-induced obese mice. EBioMedicine. (2017) 20:137–49. doi: 10.1016/j.ebiom.2017.05.028 PMC5478253 28579299

[31] Aragon-Herrera A Morana-Fernandez S Otero-Santiago M Anido-Varela L Campos-Toimil M Garcia-Seara J. The lipidomic and inflammatory profiles of visceral and subcutaneous adipose tissues are distinctly regulated by the SGLT2 inhibitor empagliflozin in Zucker diabetic fatty rats. BioMed Pharmacother. (2023) 161:114535. doi: 10.1016/j.biopha.2023.114535 36931025

[32] Billing AM Kim YC Gullaksen S Schrage B Raabe J Hutzfeldt A. Metabolic communication by SGLT2 inhibition. Circulation. (2024) 149:860–84. doi: 10.1161/CIRCULATIONAHA.123.065517 PMC10922673 38152989

[33] Herring RA Shojaee-Moradie F Stevenage M Parsons I Jackson N Mendis J. The SGLT2 inhibitor dapagliflozin increases the oxidation of ingested fatty acids to ketones in type 2 diabetes. Diabetes Care. (2022) 45:1408–15. doi: 10.2337/dc21-2043 35312749

[34] Saullo C Cruz LLD Damasceno DC Volpato GT Sinzato YK Karki B. Effects of a maternal high-fat diet on adipose tissue in murine offspring: A systematic review and meta-analysis. Biochimie. (2022) 201:18–32. doi: 10.1016/j.biochi.2022.06.009 35779649

[35] Bordeleau M Comin CH Fernandez de Cossio L Lacabanne C Freitas-Andrade M Gonzalez Ibanez F. Maternal high-fat diet in mice induces cerebrovascular, microglial and long-term behavioural alterations in offspring. Commun Biol. (2022) 5:26. doi: 10.1038/s42003-021-02947-9 35017640 PMC8752761

